# Janus Magnetic Nanoplatform for Magnetically Targeted and Protein/Hyperthermia Combination Therapies of Breast Cancer

**DOI:** 10.3389/fbioe.2021.763486

**Published:** 2022-03-08

**Authors:** Shuting Zuo, Jing Wang, Xianquan An, Yan Zhang

**Affiliations:** ^1^ Department of Breast Surgery, The Second Hospital of Jilin University, Changchun, China; ^2^ Department of Anesthesiology, The Second Hospital of Jilin University, Changchun, China

**Keywords:** combination therapies, Janus, breast cancer, magnetic targeting, nanocarriers

## Abstract

Protein therapeutics have been considered a promising strategy for cancer treatment due to their highly specific bioactivity and few side effects. Unfortunately, the low physiological stability and poor membrane permeability of most protein drugs greatly limit their clinical application. Furthermore, single-modality protein therapeutics show insufficient efficacy. To address these issues, Janus magnetic mesoporous silica nanoparticles (Janus MSNNPs) were developed to preload ribonuclease A (RNaseA) to simultaneously realize the magnetically enhanced delivery of protein drugs and magnetic hyperthermia-enhanced protein therapy. Janus MSNNPs showed a high RNaseA loading ability and pH-responsive drug release behavior. Furthermore, an external magnetic field could remarkably enhance the therapeutic effect of RNaseA-loaded Janus MSNNPs due to the improved intracellular internalization of RNaseA. Importantly, Janus MSNNPs possessed an outstanding magnetic hyperthermia conversion efficiency, which could generate hyperthermia under an alternating magnetic field, effectively supplementing protein therapy by a combined effect. *In vitro* and *in vivo* experiments confirmed the high anticancer outcome and low side effects of this intriguing strategy for breast cancer based on Janus MSNNPs. Hence, Janus MSNNPs might be an effective and safe nanoplatform for magnetically combined protein therapy.

## Introduction

Breast cancer is the most frequently diagnosed life-threatening cancer in females and the second leading cause of cancer deaths among women. An estimated million cases of breast cancer are diagnosed worldwide every year (Waks and Winer, 2019; Mckinney et al., 2020). Of all breast cancers, ∼10–20% are described as triple-negative. Triple-negative breast cancer (TNBC), which is negative for estrogen receptors, progesterone receptors and human epidermal growth factor receptor 2, is a highly malignant cancer that does not respond to hormonal therapy or specific therapy targeting HER2 receptors (Garrido-Castro et al., 2019; Yin et al., 2020). Surgery and chemotherapy are considered the standard therapies for breast cancer. Unfortunately, surgical resection only works for noninvasive TNBC, while most TNBC patients do not meet the criteria for surgery (Chang-Qing et al., 2020). Moreover, chemotherapy is restricted by severe side effects and multidrug resistance in a considerable number of TNBC patients (Pearce et al., 2017; Nedeljković and Damjanović, 2019). Thus, there is a pressing need to seek novel strategies for the efficient and safe treatment of TNBC.

Protein therapeutics, as a new mechanism for cancer therapy, have attracted great attention due to their highly specific bioactivity and few side effects (Kordalivand et al., 2018; Si et al., 2020). Unlike gene therapy, protein drugs can generate therapeutic efficacy by cell apoptosis activation, blockage of proliferation and antiangiogenesis without inducing permanent genetic alterations or adverse effects, thus holding great promise in the clinic, especially to treat chemotherapy-resistant cancers (Shao et al., 2018). Ribonuclease A (RNaseA), a relatively small protein with a size of 2.2 × 2.8 × 3.8 nm, has been applied as an alternative drug by virtue of its ability to catalyze the degradation of cellular RNA and subsequently inhibit protein production in the cytosol (Yu et al., 2017; Zhu et al., 2020). Compared with traditional chemotherapeutic drugs, RNaseA shows considerable advantages of highly specific bioactivity and avoids multidrug resistance. Although promising, the therapeutic efficacy of RNaseA is highly compromised by its low physiological stability, short half-life, and poor membrane permeability. Moreover, RNaseA alone is insufficient to control the progression of TNBC (Yu et al., 2017; Jia et al., 2019; Zhao et al., 2019). Consequently, developing a high-performance strategy for the protein therapy of TNBC is an urgent task facing the scientific community.

The combination of nanotechnology and biomedicine has allowed new approaches for cancer theranostics with high efficiency, specificity and personalization (Bjornmalm et al., 2017; Yue et al., 2018). The immobilization of proteins into nanoparticles has been shown to enhance the stability, activity and cellular internalization efficiency, leading to the development of multifunctional structures with synergistic effects as hybrid nanoplatforms (Iype et al., 2017; Xu et al., 2019). Among them, magnetic mesoporous silica nanoparticles (MMSNPs) provide good biocompatibility, high surface area and unique magnetic target properties, and thus they are considered ideal nanocarriers to achieve magnetically guided protein delivery since the cellular uptake and tumor accumulation of MMSNPs can be improved by using an external magnetic field (Chang et al., 2018). More importantly, M-MSNs can produce hyperthermia under an alternating current magnetic field (ACMF), termed magnetic hyperthermia therapy, a noninvasive treatment, which can effectively supplement the antitumor efficacy of RNaseA (Xing et al., 2018). Therefore, MMSNPs might be an excellent candidate for the protein therapy of TNBC. Nevertheless, the potential of magnetically enhanced RNaseA therapy by MMSNPs has not yet been explored.

**SCHEME 1:**
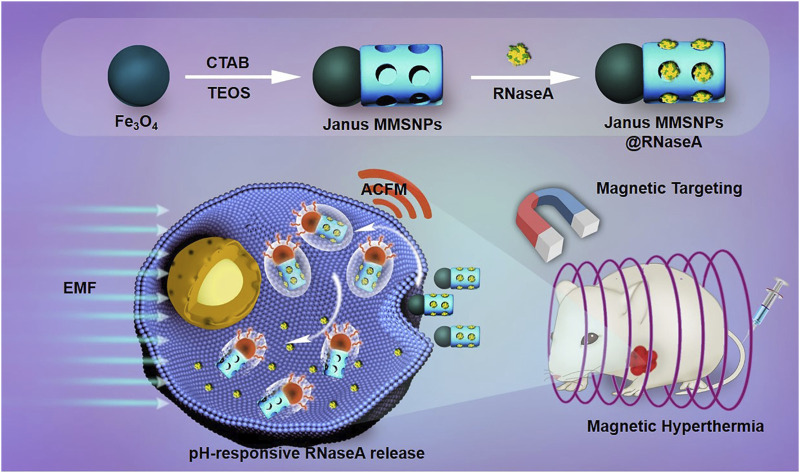
Schematic illustration of the preparation of RNaseA-loaded Janus MMSNPs and their application for magnetically targeted and protein/hyperthermia combination therapies of breast cancer.

Janus nanomaterials, named after the double-faced Roman mythology god, are compartmentalized particles with two sides of different chemical compositions, and they have gained considerable interest due to their noninterfering properties on anisotropic surfaces (Zhang et al., 2020; Shao et al., 2021). Janus metallic mesoporous silica nanocarriers have been demonstrated to have unique optical, magnetic, thermal, and electric properties in comparison to the conventional core-shell structures, thus showing a potentially synergistic and stimuli-responsive manner in combined diagnosis and therapy of cancer (Hernández-Montoto et al., 2019; Zhang et al., 2019; Xing et al., 2020). In this work, Janus MMSNPs were developed using a sol-gel method and functionalized with carboxyl groups for RNaseA loading and pH-responsive release. Janus MMSNPs showed a high RNaseA loading efficiency, magnetic response performance and magnetic hyperthermia conversion efficiency. Then, we investigated their performance in magnetically targeted and hyperthermia-enhanced suicide protein therapy *in vitro* and *in vivo*. In light of the good therapeutic outcome and biosafety, Janus MMSNPs might be a promising platform for efficient protein therapy of TNBC (Scheme 1).

## Methods

### Preparation of Janus MMSNPs

Polyacrylic acid (PAA)-stabilized Fe_3_O_4_ NPs were fabricated using a high-temperature hydrolysis reaction. Polyacrylic acid (PAA)-stabilized Fe_3_O_4_ NPs were fabricated using a high-temperature hydrolysis reaction. Briefly, 0.13 mg FeCl_3_ was dissolved into the 34 ml of diethylene glycol under the stirring at 30°C. At the same time, 2.0 g NaOH was dissolved into the 20 ml of diethylene glycol with stirring at 120°C. After 1 h, the diethylene glycol solution of FeCl_3_ was heated to 265°C for 30 min under the protection of nitrogen gas. Then, 3.4 ml diethylene glycol solution of NaOH was added into the diethylene glycol solution of FeCl_3_ and reacted at 265°C for 1 h. Then, the polyacrylic acid (PAA)-stabilized Fe_3_O_4_ NPs were obtained after washed with ethyl alcohol and water and centrifugation. Then, Janus MMSNPs were fabricated by a modified sol-gel method. Briefly, 1 ml of Fe_3_O_4_ NP solution (8.4 mg/ml) was mixed with 10 ml of CTAB solution (5 mg/ml) under ultrasonication for 30 min. Then, 40 μL of tetraethyl orthosilicate (TEOS) was added dropwise into the mixture, followed by 0.5 ml of ammonium hydroxide (14.8 M). After 20 min of stirring at 40°C, the products were collected by magnetic separation using a magnet and washed with deionized water and ethyl alcohol three times. To form a mesoporous structure, the prepared products were dispersed in a NH_4_NO_3_ solution and refluxed for 12 h to extract CTAB. For RNaseA loading and PEG conjugation, carboxylate groups were introduced on the surface of Janus MMSNPs. First, Janus MMSNPs were modified with -NH_2_ groups using ammonium persulfate (APS) through postgrafting according to a previous report. Then, 20 mg of Janus MMSNPs was dispersed in a DMF solution of succinic anhydride (100 ml, 2 wt%) and stirred overnight at the room temperature. The obtained Janus MMSNPs with -COOH functionalization were collected and washed. Finally, PEG was conjugated onto the Janus MMSNPs through an EDC/NHS reaction. Briefly, the Janus MMSNP suspension was mixed with an EDC/NHS aqueous solution and sonicated for 30 min. Then, PEG was added into the mixture with stirring for 24 h. Finally, PEG-grafted Janus MMSNPs were collected and stored at 4°C for further use.

### RNaseA Loading and Release

20 mg of RNaseA was added into 10 ml of deionized water to prepare a protein stock solution. Then, 20 ml of Janus MMSNPs (2 mg/ml) was mixed with 10 ml of the prepared protein stock solution, and the mixture was stirred at 4°C for 24 h. After centrifugation, the loading amount of RNaseA was determined by detecting the absorbance of the supernatant at 280 nm and calculated using the following equation: Loading content (%) = Mass of drug in NPs/Mass of drug-loaded NPs. For the RNaseA release test, 5 mg of RNaseA-loaded Janus MMSNPs was encapsulated into a dialysis bag and placed in 10 ml of PBS solutions with different pH values (pH = 5.5 and 7.4). Then, the amount of RNaseA released was detected at predetermined time points by the UV-vis spectrometry.

### Intracellular Internalization and Drug Release

MCF-7 cells were obtained from ATCC and cultured at 37°C in RPMI 1640 medium with 10% fetal bovine serum penicillin and streptomycin. MCF-7 cells were seeded in a 6-well plate and maintained overnight. To detect the cellular uptake of Janus MMSNPs, FITC-labeled Janus MMSNPs were incubated with MCF-7 cells at a concentration of 12.5 μg/ml for 6 h. For the EMF-treated groups, small NdFeB permanent magnets (surface magnetic field of 0.2 T) were placed on the bottom of the 6-well plate for 30 min. Then, the cells were stained with Hoechst 33258 and LysoTracker Red DND-99 (Invitrogen) for observation by CLSM. To quantify the intracellular internalization of Janus MMSNPs, the cells were washed, trypsinized and resuspended after the same treatment with Janus MMSNPs and measured by FACS. To investigate the intracellular release of RNaseA, RNaseA was labeled with Cy5.5 and preloaded into Janus MMSNPs. Then, RNaseA-loaded Janus MMSNPs were incubated with MCF-7 cells for 6 h and detected by CLSM and FACS using the same protocol.

### *In vitro* Therapeutic Effect of Janus MMSNPs@RNaseA

MCF-7 cells were seeded in a 96-well plate and cultured overnight. Then, these cells were treated with various concentrations of Janus MMSNPs, RNaseA and Janus MMSNPs@RNaseA for 24 h with or without 30 min of external magnetic field (EMF) exposure. For the EMF treatment groups, a small NdFeB permanent magnets (0.2T) were placed under the 96-well plates during the incubation of 0.5 h. Notably, the amount of RNaseA in Janus MMSNPs@RNaseA was equal to that of free RNaseA in the same parallel groups. Then, cell viability was assessed using WST assays, and untreated cells were used as the control group.

To investigate the magnetic hyperthermia conversion efficacy of Janus MMSNPs, cell culture medium containing various concentrations of Janus MMSNPs@RNaseA was placed and exposed to an alternating magnetic field (ACFM, 325 Oe, 262 kHz), and the temperature change was monitored at predetermined time points. To investigate the magnetic hyperthermia therapy of Janus MMSNPs, MCF-7 cells were treated with various concentrations of Janus MMSNPs with or without an ACFM (325 Oe, 262 kHz) in the absence or presence of an EMF. For the ACFM treatment groups, the cells were exposed in an ACFM for 30 min during the incubation of 6 h. After 24 h of incubation, the cell viability was measured using the same method.

### *In vivo* Combined Therapies and Biosafety

All animal experiments were approved by the Ethics Committee for the Use of Experimental Animals of Jilin University. To establish MCF-7 tumor-bearing nude mice, 5 × 10^6^ MCF-7 cells were orthotopically injected into the mammary fat pads of female nude mice weighing approximately 20 g. When the volume of these tumors grew to approximately 80 mm^3^, all of the mice were separated into six groups and intravenously administered PBS, free RNaseA (5 mg/kg), Janus MMSNPs (20 mg/kg) and Janus MMSNPs@RNaseA (25 mg/kg) every 3 days. For the EMF-treated groups, a NdFeB permanent magnet (surface magnetic field of 0.5 T) was placed on the skin over the tumor site for 1 h at 1 h after administration. For the ACMF-treated groups, the mice were placed under an alternating magnetic field (325 Oe, 262 kHz) for 1 h at 24 h after administration. The tumors and body weight in each group were recorded every 3 days. Tumor volumes were caculated using the equation: tumor volume = the length of tumor × the width of tumor × the width of tumor ^2^ × 0.52. All the mice were sacrificed at the 23rd days. The tumors were harvested and weighed. The liver, spleen, kidneys, lungs and heart were harvested, sliced and stained with hematoxylin-eosin. The serum was separated from the collected blood, and the serum biochemical indices, including aminotransferase (AST), phosphocreatine kinase (CK), alanine aminotransferase (ALT), total bilirubin (TBIL), alkaline phosphatase (ALP), blood urea nitrogen (BUN), creatinine (CR), cholesterol (TC) and triglyceride (TG) were assessed using the manufacturer’s protocol.

## Results and Discussion

**FIGURE 1:**
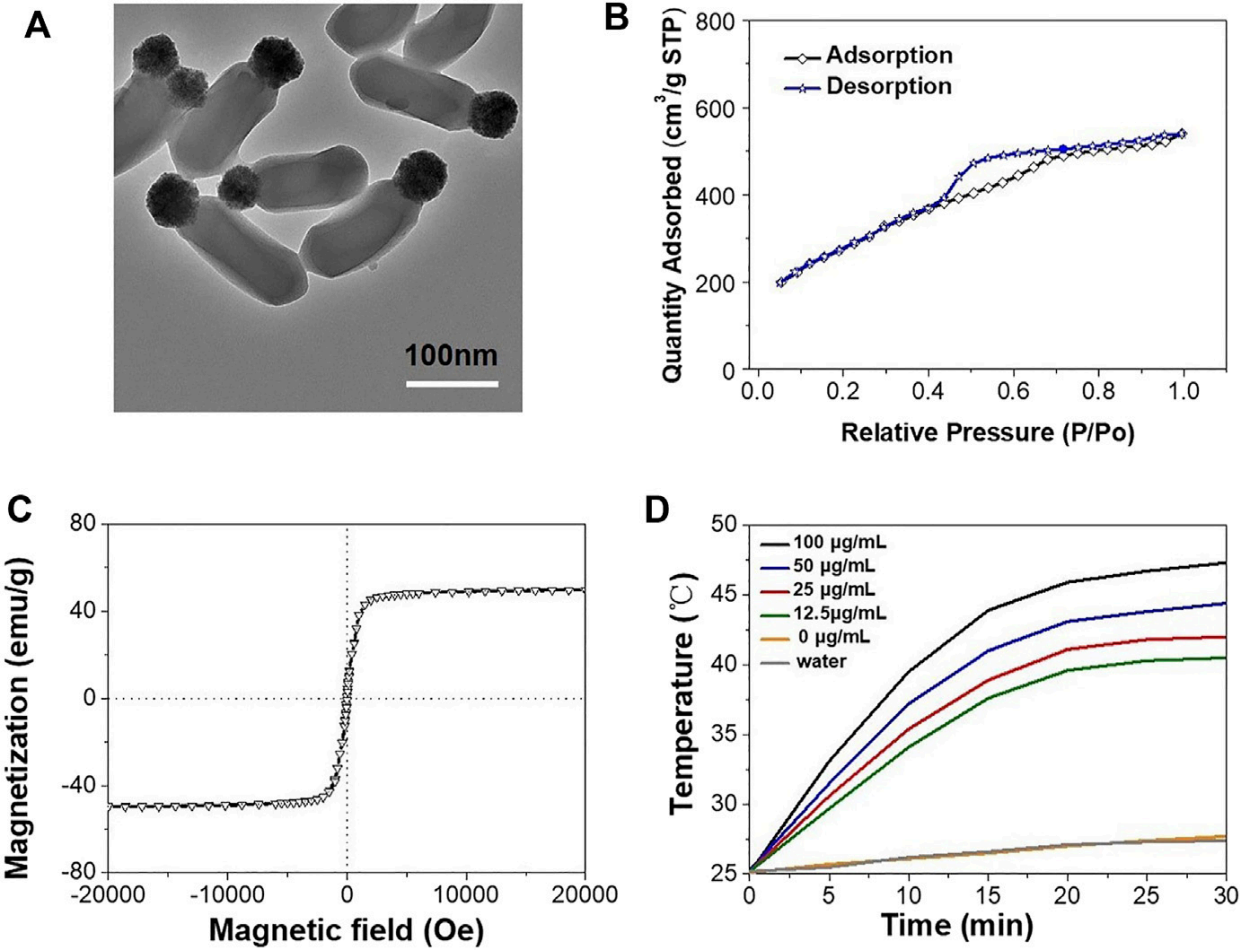
Characterization of Janus MMSNPs. **(A)** TEM images of Janus MMSNPs, scale bar = 100 nm. **(B)** N_2_ adsorption-desorption isotherms of Janus MMSNPs. **(C)** Magnetization curve of Janus MMSNPs. **(D)** Temperature-time curves of various concentration of Janus MMSNPs solutions.

Janus MMSNPs were constructed using a sol-gel method, in which Fe_3_O_4_ nanoparticles were used as a substrate, TEOS was used as a silica source, and CTAB was used as a template. Transmission electron microscopy (TEM) in Figure 1A clearly revealed that Janus MMSNPs showed a uniform morphology and good monodispersity, which consisted of a Fe_3_O_4_ head with a size of approximately 50 nm and a silica body with dimensions of approximately 150 nm. Subsequently, Janus MMSNPs were modified with carboxyl groups and conjugated with polyethylene glycol (PEG) amine. After PEG decoration, Janus MMSNPs showed long-term stability in cell medium for 5 days of storage (Supplementary Figure S1). Then, we investigated the magnetic properties of these Janus MMSNPs. As shown in the Figure 1C, Janus MMSNPs possess good magnetic properties with a saturation magnetization value of 49 emu·g^−1^. We next investigated the mesoporous properties of Janus MMSNs. The N_2_ adsorption-desorption isotherm curve in Figure 1B indicated that Janus MMSNPs have a large pore volume (0.57 cm^3^/g), a high BET surface area (578.2 m^2^/g) and a uniform pore diameter (4.2 nm). The excellent mesoporous properties of Janus MMSNs suggested a high drug loading efficiency. To investigate the magnetic hyperthermia conversion ability of Janus MMSNPs, we measured the temperature change of cell culture media containing various concentrations of Janus MMSNPs under an alternating current magnetic field (ACMF). As shown in Figure 1D, the temperature of pure water and cell culture medium without Janus MMSNPs only increased by 6°C under an ACMF, whereas the temperature increase in Janus MMSNP solutions was in a time- and concentration-dependent manner with the exposure to ACMF. At a Janus MMSNP concentration of 50 μg/ml after less than 20 min of exposure to ACMF, the temperature of the cell medium rose to 43°C, a crucial temperature to induce cancer cell death.

**FIGURE 2:**
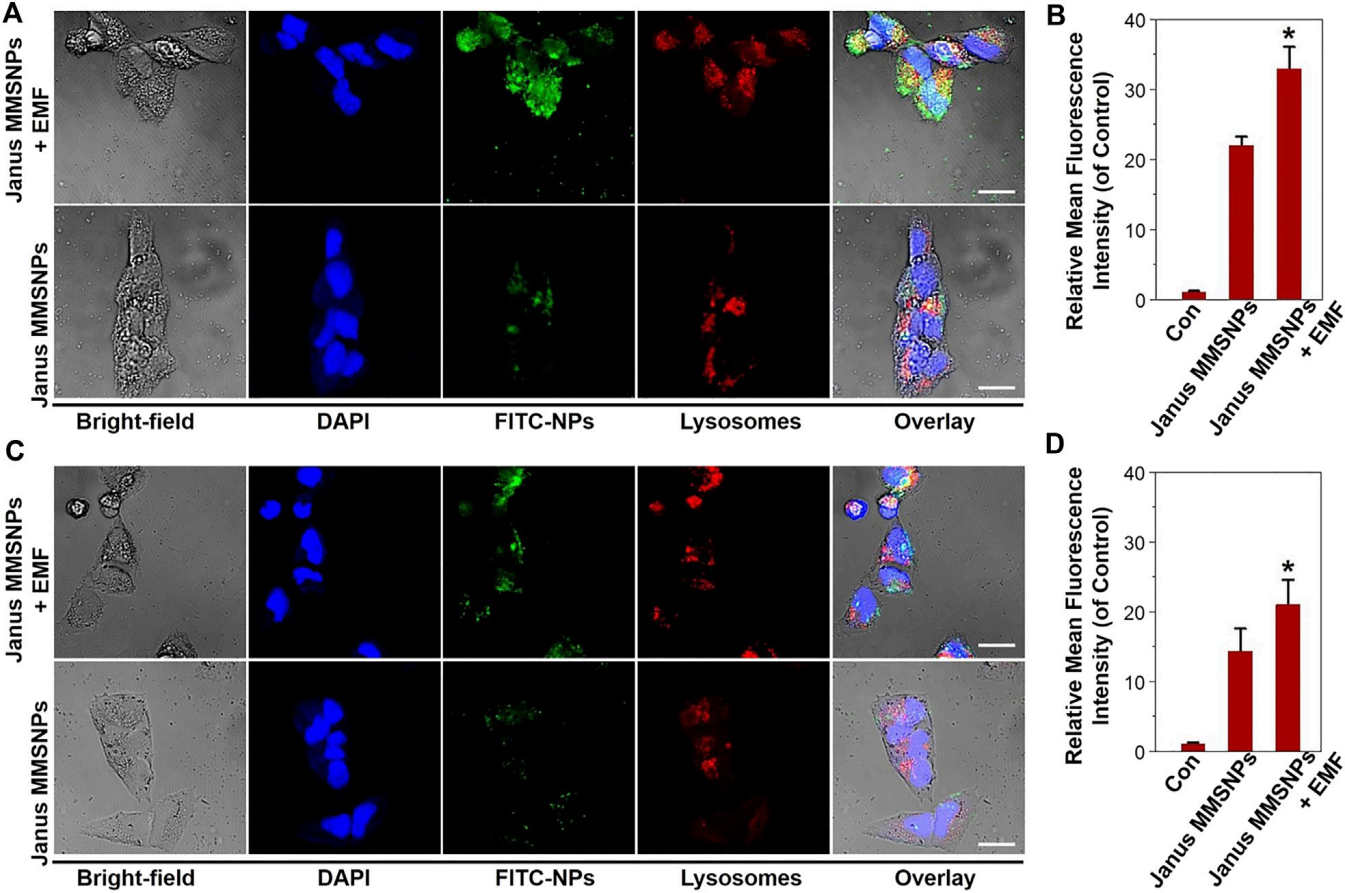
Cellular uptake of Janus MMSNPs. CLSM images of MCF-7 cells **(A)** and MCF 10A cells **(C)** incubated with Janus MMSNPs for 6 h with or without an EMF, scale bars = 10 μm. Quantitative FACS analysis of the endocytosis of Janus MMSNPs in MCF-7 cells **(B)** and MCF 10A cells **(D)** after 6 h of incubation with or without an EMF. Mean values ± SD. *p < 0.05 vs. the Janus MMSNPs group, *n* = 3.

Numerous studies have demonstrated that an external magnetic field (EMF) could improve the cellular uptake of magnetic nanocarriers and direct their transportation to tumor sites, which was also named as “magnetic targeting”. Thus, we investigated the cellular internalization of Janus MMSNPs under a magnetic field stimulus. As shown in Figures 2A, C, the colocalization of FITC-labeled Janus MMSNPs with endolysosomes was observed after 6 h of incubation with MCF-7 cells and MCF 10A cells with or without an EMF by CLSM, indicating that the Janus MMSNPs could enter into the cells and internalize in the endolysosomes. As expected, higher fluorescence signals were found in the magnetic field treated groups than that those of EMF-untreated groups, which was in line with the FACS results (Figures 2B, D), confirming the utility of the EMF to promote the cellular internalization of Janus MMSNPs. Notably, more Janus MMSNPs distributed in MCF-7 cells compared with MCF 10A cells at the same EMF-treated condition, indicated that Janus MMSNP could induce a higher cellular uptake in cancer cells than normal cells.

**FIGURE 3:**
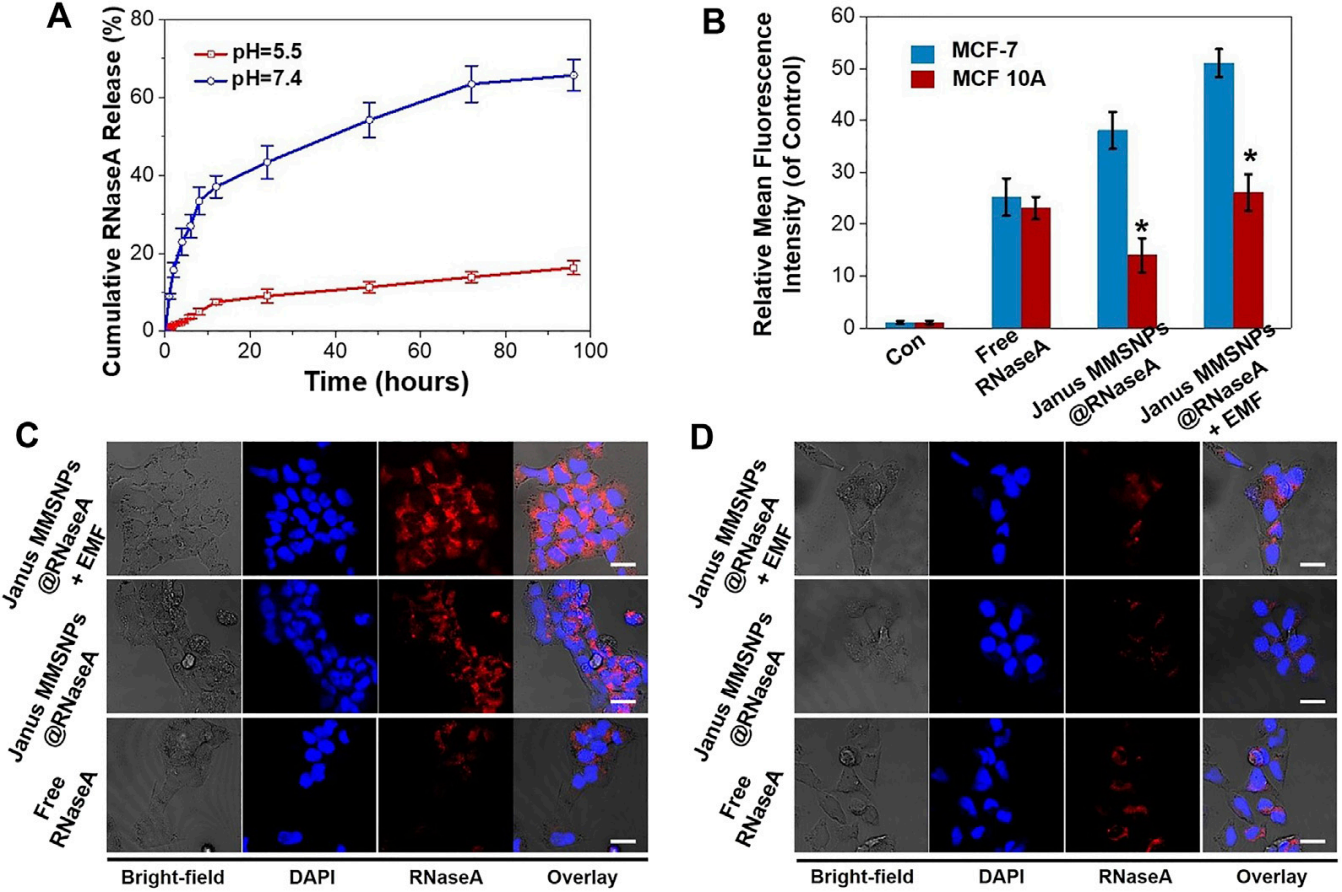
Drug release behavior of Janus MMSNPs@RNaseA. **(A)** Drug-release behavior of Janus MMSNPs@RNaseA at different pH value. **(B)** Quantitative FACS analysis of the intracellular distribution of RNaseA in free RNaseA-, Janus MMSNPs@RNaseA -, or Janus MMSNPs@RNaseA plus an EMF-treated MCF-7 or MCF 10A cells for 6 h. Mean values ± SD. *p < 0.05 vs. the MCF-7 cell group, *n* = 3. **(C)** CLSM images of MCF-7 cells after treated with free RNaseA or Janus MMSNPs@RNaseA with or without an EMF for 6 h **(D)** CLSM images of MCF cells after treated with free RNaseA or Janus MMSNPs@RNaseA with or without an EMF for 6 h. Scale bar = 10 μm.

To explore the potential for protein delivery, the carboxyl-functionalized Janus MMSNPs were loaded with Ribonuclease A (RNaseA), a cytotoxic protein drug commonly used in the breast cancer therapy. The zeta potential change indicated that RNaseA was successfully loaded into the Janus MMSNPs (Supplementary Figure S3). As determined by UV-visible spectrophotometry, the drug loading content of RNaseA was as high as 250.6 μg mg^−1^. To investigate the drug release behavior, the cumulative release of RNaseA from RNaseA-loaded Janus MMSNs (Janus MMSNs@RNaseA) was measured in PBS solution at different pH values (5.5 and 7.4). As shown in Figure 3A, less than 20% of RNaseA was released from Janus MMSNs@RNaseA after 96 h of dialysis at a pH of 7.4, whereas a rapid release of RNaseA occurred at a pH of 5.5, and the cumulative release reached 66.1% at 96 h. The pH-responsive drug release phenomenon could be explained by RNaseA becoming more water soluble at low pH because of the protonation of amine groups. Since endolysosomes and the local environment of tumor tissues are acidic, pH-responsive drug release behavior can improve the anticancer effect and reduce the side effects in normal tissues. To investigate the intracellular drug release, RNaseA was labeled with Cy 5.5, and the red fluorescence signals of RNaseA were detected in MCF-7 cells and MCF-10A cells after 6 h of incubation using CLSM and FACS. As shown in Figures 3B–D, more green fluorescent signals were detected in MCF-7 cells after treated with Janus MMSNPs@RNaseA than those treated with free RNaseA, indicating Janus MMSNPs are effective carriers to improve the cellular internalization of RNaseA in cancer cells. On the contrary, Janus MMSNPs@RNaseA groups showed a lower intracellular RNaseA distribution than free RNaseA, which could be attributed to the weak cellular internalization of Janus MMSNPs and the low RNaseA release in normal cells. Additionally, an external magnetic field obviously improved the RNaseA accumulation in both MCF-7 cells and MCF 10A cells after treated with Janus MMSNPs@RNaseA. Other studies have demonstrated that in comparison to the core-shell nanoparticles, Janus magnetic nanoparticles have a higher cellular uptake efficiency due to the rod-like morphology and stronger magnetic responsive property because of the non-interfering feature (Shao et al., 2016). The magnetically-enhanced performance indicated that Janus MMSNPs might be a superior nanoplatform for protein therapies of cancer.

**FIGURE 4:**
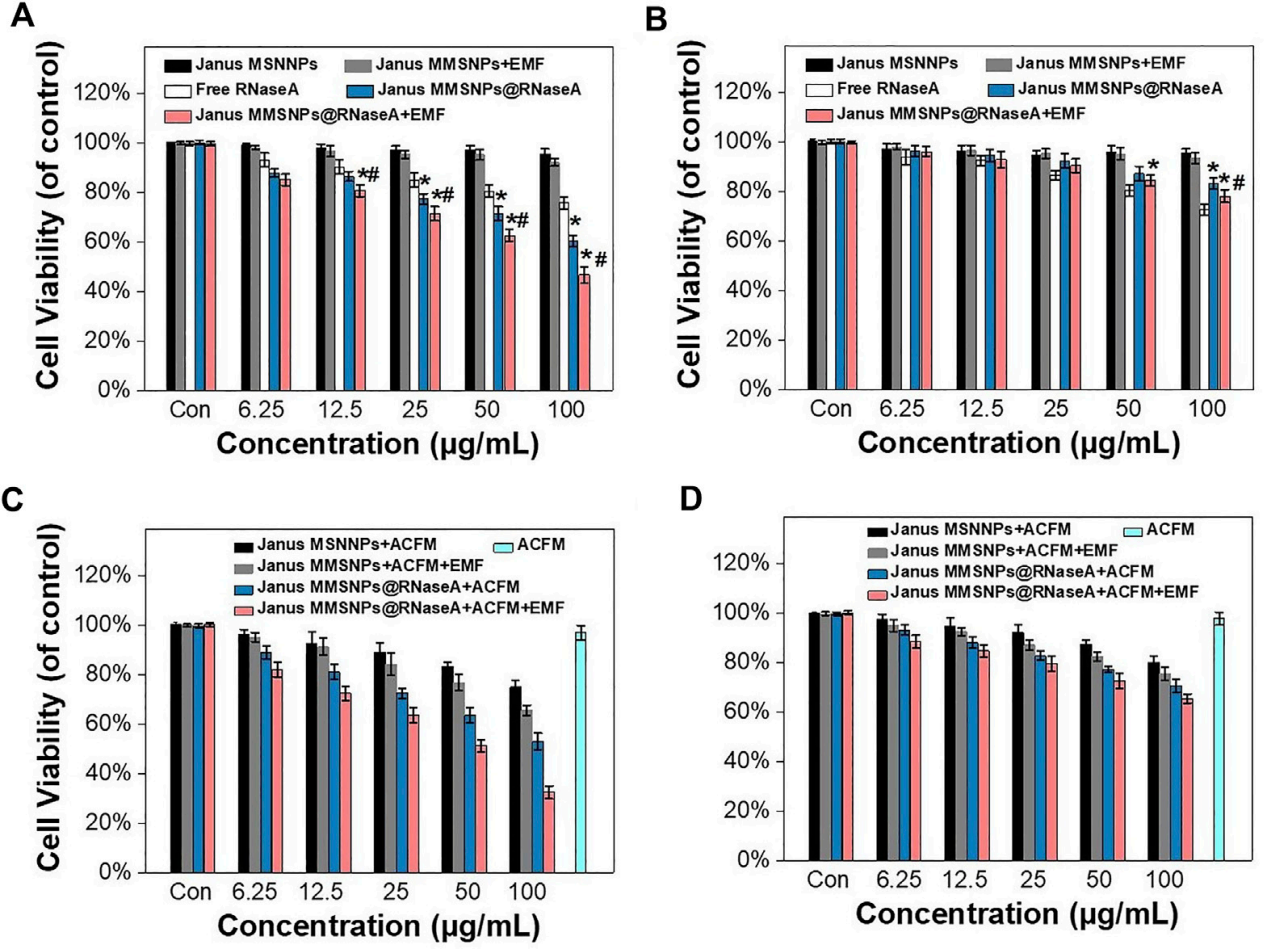
Combination therapies of Janus MMSNPs@RNaseA *in vitro*. Cell viability of **(A)** MCF-7 and **(B)** MCF 10A after incubation with free RNaseA, Janus M-MSNs or Janus MMSNPs@RNaseA with or without an EMF for 24 h *p < 0.05 vs. free RNaseA group; ^#^ *p* < 0.05 vs. Janus MMSNPs@RNaseA group. The cytotoxicity of Janus MMSNPs@RNaseA in the absence or presence of an ACMF with or without EMF towards **(C)** MCF-7 cells and **(D)** MCF 10A for 24 h. Mean values ± SD, *n* = 4.

Endocytosis and drug distribution play important roles in the therapeutic effect. The WST assay was subsequently performed to evaluate the cytotoxicity of Janus MMSNPs@RNaseA with the aim of an EMF. As shown in Figures 4A,B, Janus MMSNPs showed a negligible cytotoxicity towards MCF-7 cells or MCF 10A cells even at a concentration of 100 μg/ml with or without an EMF, indicating that Janus MMSNPs have a good biocompatibility. The anticancer effect of Janus MMSNPs@RNaseA and free RNaseA was concentration-dependent in MCF-7 cells. Janus MMSNPs@RNaseA showed a higher killing effect towards MCF-7 cells than free RNaseA when the amount of RNaseA was equal, whereas Janus MMSNPs@RNaseA induced lower death of MCF 10A cells than free RNaseA, which was in line with the results of intracellular RNaseA accumulation. These results indicated the anticancer efficacy of Janus MMSNPs@RNaseA originated from the selective endocytosis and pH-responsive drug release in cancer cells rather than in normal cells. Notably, the applied EMF remarkably improve the therapeutic efficiency of Janus MMSNPs@RNaseA, which was due to the magnetically enhanced endocytosis. Given the ability of Janus MMSNPs to generate magnetic hyperthermia, we investigated the therapeutic effect of magnetic hyperthermia on Janus MMSNP-treated MCF-7 cells. As shown in Figures 4C,D, the cell viability of MCF-7 cells and MCF 10 A cells was not decreased only after exposure to ACMF in the absence of Janus MMSNPs, indicating the biosafety of the applied ACMF. As expected, Janus MMSNPs plus ACMF showed a concentration-dependent anticancer effect, and the therapeutic effect of magnetic hyperthermia could be improved by an EMF due to magnetically enhanced endocytosis. We subsequently explored the combined effect of magnetic hyperthermia therapy with protein therapy. The Figures 4C,D revealed that Janus MMSNPs@RNaseA plus ACMF could induce more deaths of MCF-7 cells than single Janus MMSNPs@RNaseA or Janus MMSNPs plus ACMF. Additionally, Janus MMSNPs@RNaseA plus ACMF with EMF showed the best anticancer effect among all the treatment groups. These results indicated that both ACMF and EMF could improve the outcome of protein therapy and that the combination of these two stimuli could result in the best therapeutic efficacy.

**FIGURE 5:**
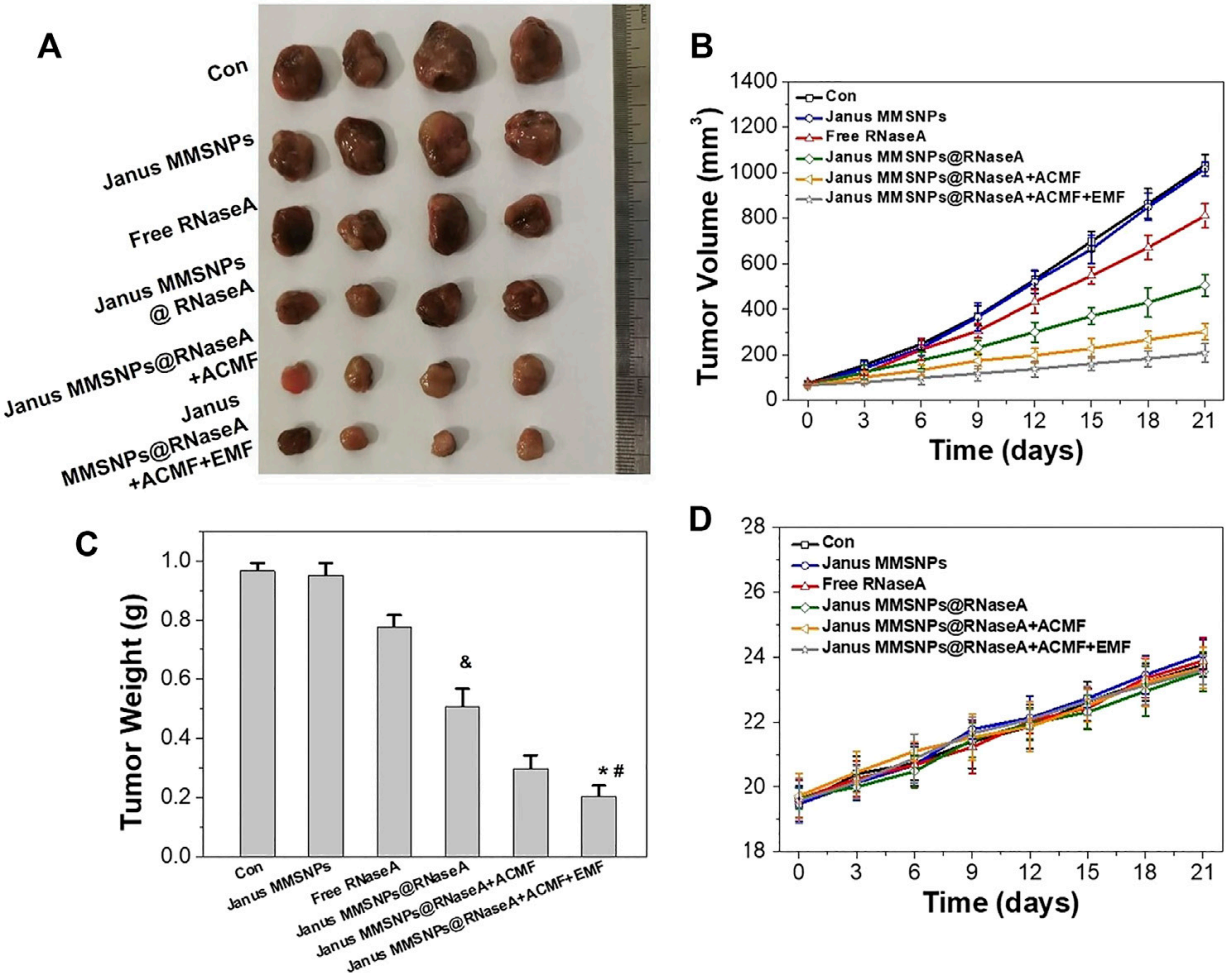
*In vivo* combined protein/magnetic hyperthermia therapes of Janus MMSNPs@RNaseA. **(A)** Tumor photographs. **(B)** Tumor growth curves. **(C)** Tumor weights Mean values ± SD, *n* = 4. *p < 0.05 vs. the Janus MMSNPs@RNaseA, ^#^ *p* < 0.05 vs. Janus MMSNPs@RNaseA plus ACFM, and ^&^ *p* < 0.05 vs. the free RNaseA groups. **(D)** Body weight change.

After confirming the Janus MMSNPs@RNaseA-based combination therapies *in vitro*, we further investigated magnetically enhanced protein therapy *in vivo*. MCF-7 tumor-bearing nude mice were established and randomly divided into six groups. As shown in Figures 5A–C, tumors in the Janus MMSNP-treated groups grew similarly to those in the control groups, indicating that Janus MMSNPs have no inhibitory effect on tumor growth. Janus MMSNPs@RNaseA showed delayed growth in the relative tumor volumes and tumor weights compared with the control groups or the free RNaseA groups. Additionally, the inhibitory rate of tumor growth was remarkably higher in the Janus MMSNPs@RNaseA plus ACMF groups than in the Janus MMSNPs@RNaseA groups, and Janus MMSNPs@RNaseA plus ACMF with EMF showed the highest therapeutic efficacy among all of the treatment groups, indicating the magnetically enhanced combination therapy based on our Janus MMSNPs@RNaseA.

**FIGURE 6:**
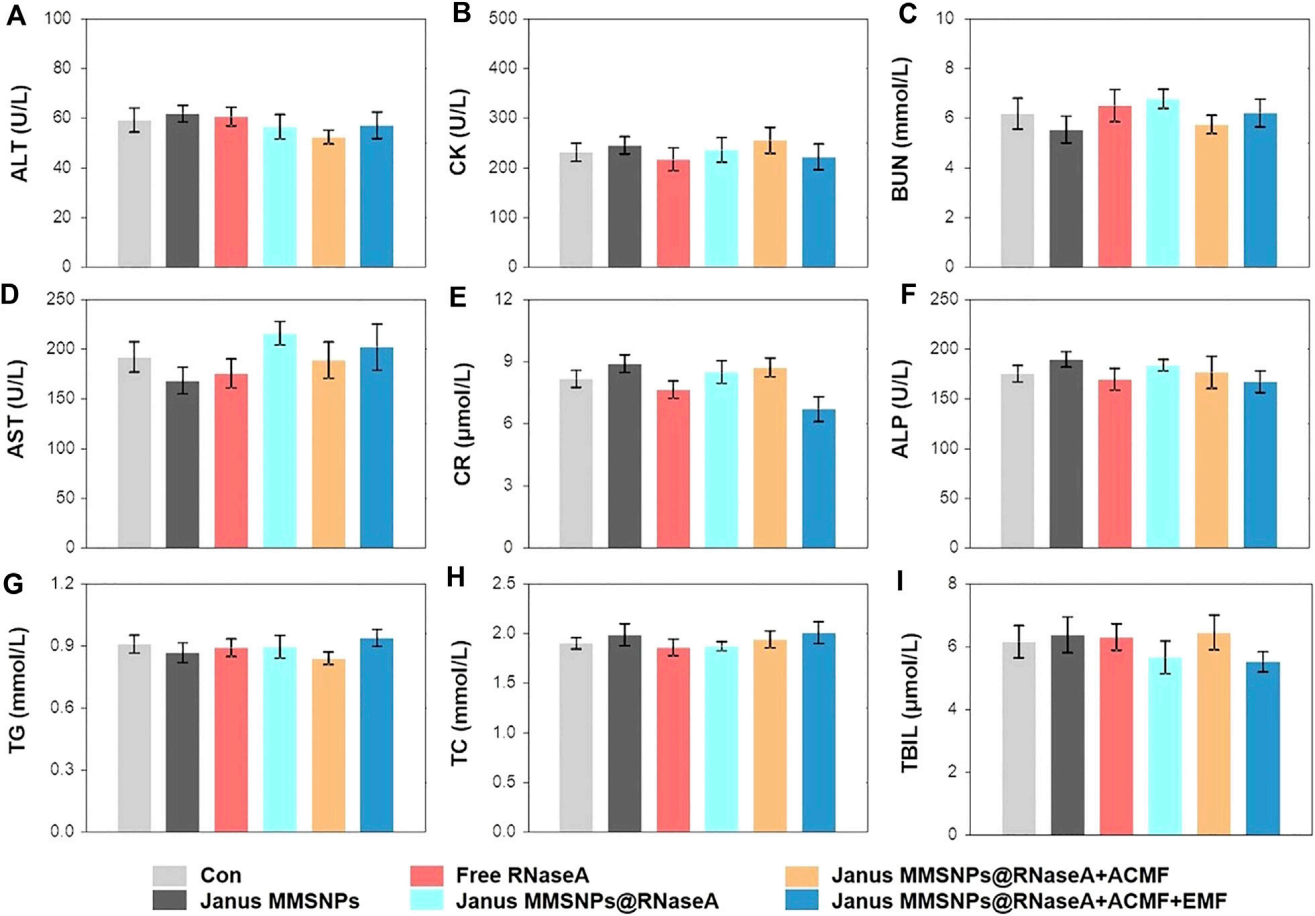
Serum biochemistry indicators assessment. **(A)** Alanine aminotransferase (ALT). **(B)** Phosphocreatine kinase (CK). **(C)** Blood urea nitrogen (BUN). **(D)** Aspartate aminotransferase (AST). **(E)** Creatinine (CR). **(F)** Alkaline phosphatase (ALP). **(G)** Triglyceride (TG). **(H)** Cholesterol (TC). **(I)** Total bilirubin (TBIL) of MCF-7 tumor-bearing nude mice. Mean values ± SD, *n* = 4.

**FIGURE 7:**
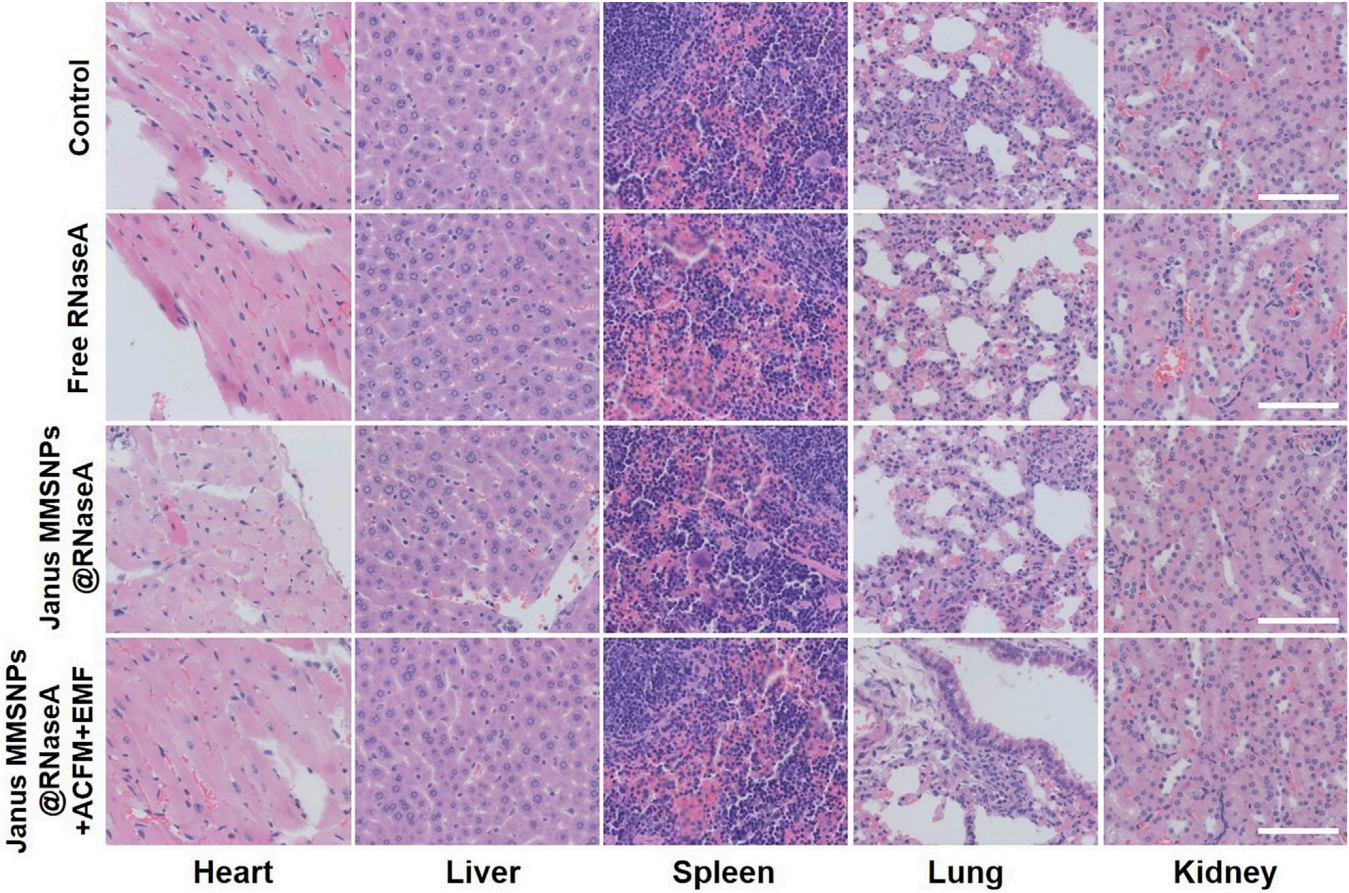
Effect of protein/hyperthermia combination therapies on the histopathology of heart, liver, spleen, lung and liver from MCF-7 tumor bearing nude mice. Scale bar = 100 μm.

Encouraged by the outstanding therapeutic efficacy, we evaluated the systemic toxicity of the combination therapies. As shown in Figure 5D and Figure 6, none of the mice experienced weight loss, and no observable changes occurred in serum biochemical indices, including aspartate aminotransferase (AST), phosphocreatine kinase (CK), alkaline phosphatase (ALP), alanine aminotransferase (ALT), blood urea nitrogen (BUN), creatinine (CR), total bilirubin (TBIL), cholesterol (TC) and triglyceride (TG), after being subjected to a full course of any treatments. Furthermore, hematoxylin-eosin staining images of the heart, liver, spleen, lungs and kidneys in Figure 7 indicated a normal physiology of all of the mouse organs after receiving any treatment. Collectively, consistent with our *in vitro* results, Janus MMSNPs@RNaseA exposed to an EMF displayed a magnetically enhanced protein therapeutic outcome *in vivo* with negligible side effects.

## Conclusion

In summary, Janus MMSNPs were fabricated to preload RNaseA for realizing magnetically enhanced protein therapy of breast cancer. The prepared Janus MMSNPs showed a high RNaseA loading efficiency, pH-responsive protein release property and improved protein delivery with the help of an external magnetic field. Importantly, the therapeutic effect of Janus MMSNPs could be promoted by magnetically enhanced tumor accumulation and their combination with magnetic hyperthermia therapy. Given their good magnetic targeting abilities and the remarkable combined effect of magnetic hyperthermia therapy with protein therapy, Janus MMSNPs might be a potentially superior candidate for the protein therapy of breast cancer.

## Data Availability

The original contributions presented in the study are included in the article/Supplementary Material, further inquiries can be directed to the corresponding author.
