# Supplementary material for: Janus Magnetic Nanoplatform for Magnetically Targeted and Protein/Hyperthermia Combination Therapies of Breast Cancer
Source: Front Bioeng Biotechnol. 2022 Mar 8;9:763486. doi: 10.3389/fbioe.2021.763486 (PMC8958000; doi:10.3389/fbioe.2021.763486)
Supplement: Supplementary file 1 [file DataSheet1.docx]

Supplementary Material

Shuting Zuo ^1^, Jing Wang ^1^, Xianquan An ^2^, Yan Zhang^1^*

^1^Department of Breast Surgery, The Second Hospital of Jilin University, Changchun, PR China.

^2^Department of anesthesiology, The Second Hospital of Jilin University, Changchun, PR China.

*** Correspondence:**Corresponding Author
zhangy01@jlu.edu.cn

# Materials

Tetraethyl orthosilicate (TEOS), 3-aminopropyltriethoxysilane (APTES), polyacrylic acid (PAA, 8 mmol, Mw = 1800), iron (III) chloride anhydrous (FeCl_3_), diethylene glycol (DEG), cetyltrimethyl ammonium bromide (CTAB) and fluorescein isothiocyanate(FITC) were obtained from Sigma-Aldrich Co. (St Louis, MO, USA). RPMI-1640 medium and fetal bovine serum (FBS) were bought from GIBCO. Diagnostic kits for an aspartate aminotransferase (AST), phosphocreatine kinase (CK), alkaline phosphatase (ALP), alanine aminotransferase (ALT), blood urea nitrogen (BUN), creatinine (CR), total bilirubin (TBIL), cholesterol (TC) and triglyceride (TG) were purchased from Nanjing Jiancheng Bioengineering Institute. All reagents were commercial products and could be used without further purification.


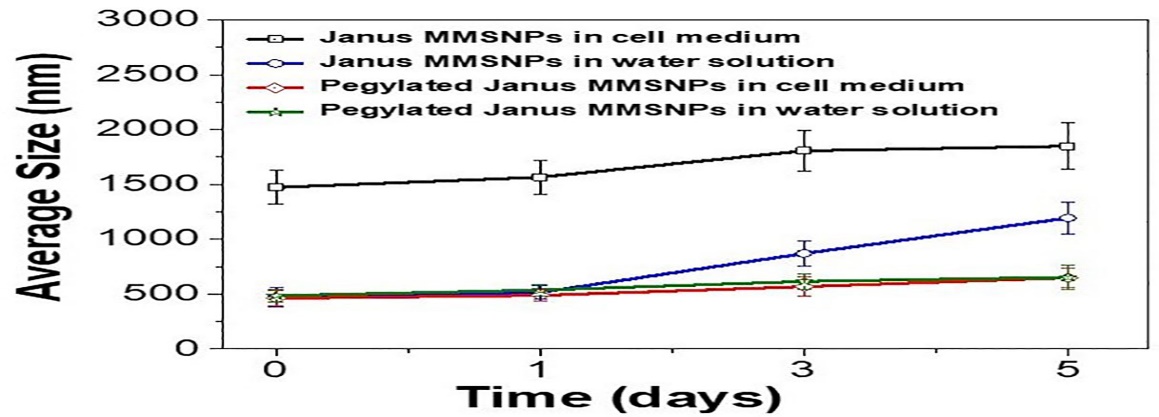


**Supplementary Figure 1.** Size distribution of Janus MMSNPs after stockage in water or cell medium for 5 days.


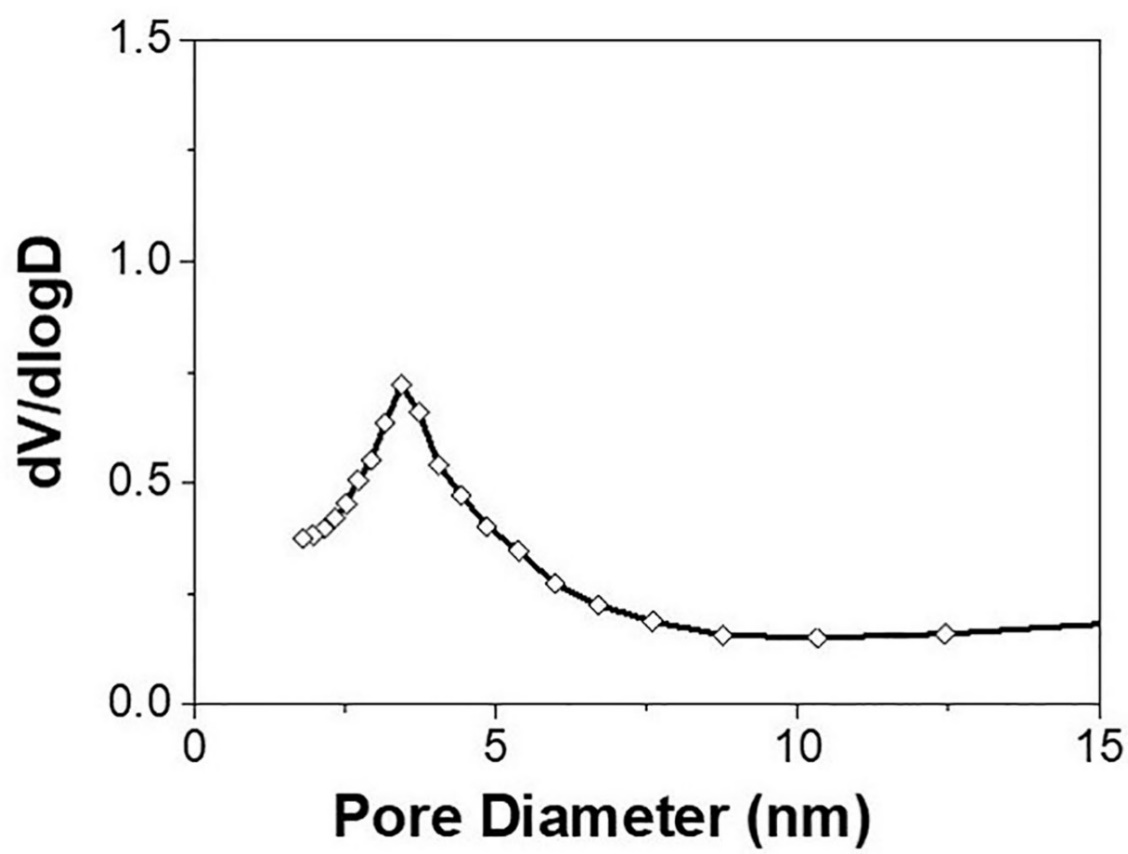


**Supplementary Figure 2.** Pore size distribution of Janus MMSNPs.


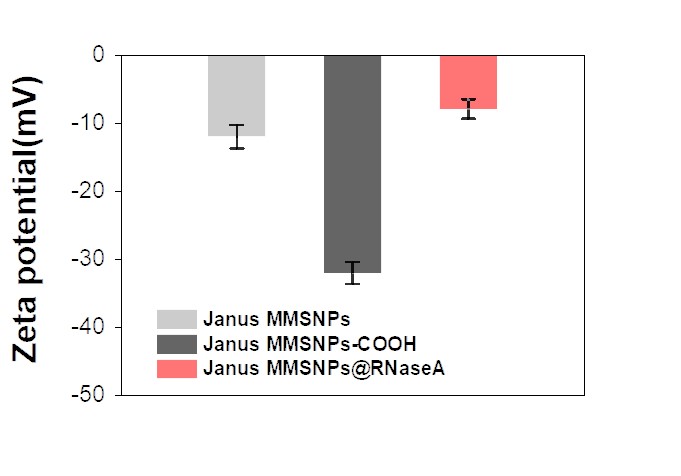


**Supplementary Figure 3.** Zeta potential of Janus MMSNPs, carboxyl-functionalized Janus MMSNPs (Janus MMSNPs-COOH) and Janus MMSNPs@RNaseA.
